# Nutritional immunology: function of natural killer cells and their modulation by resveratrol for cancer prevention and treatment

**DOI:** 10.1186/s12937-016-0167-8

**Published:** 2016-05-04

**Authors:** Christian Leischner, Markus Burkard, Matthias M. Pfeiffer, Ulrich M. Lauer, Christian Busch, Sascha Venturelli

**Affiliations:** 1 Department of Internal Medicine I, Medical University Hospital, Otfried-Mueller-Str. 27, Tuebingen, Germany; 2 Division of Dermatologic Oncology, Department of Dermatology and Allergology, University of Tuebingen, Tuebingen, Germany; 3 Department of Pediatric Hematology and Oncology, University Children’s Hospital, Tuebingen, Germany; 4 Pallas Clinic, Olten, Switzerland

**Keywords:** Nutritional immunology, Resveratrol, Immune modulation, NK cell activity, Innate immune system

## Abstract

Natural killer (NK) cells as part of the innate immune system represent the first line of defence against (virus-) infected and malignantly transformed cells. The emerging field of nutritional immunology focuses on compounds featuring immune-modulating activities in particular on NK cells, which e.g. can be exploited for cancer prevention and treatment. The plant-based nutrition resveratrol is a ternary hydroxylated stilbene, which is present in many foods and beverages, respectively. In humans it comprises a large variety of distinct biological activities. Interestingly, resveratrol strongly modulates the immune response including the activity of NK cells. This review will give an overview on NK cell functions and summarize the resveratrol-mediated modulation thereof.

## Background

The innate immune system is conserved among vertebrates and is already functionally present at birth. Cellular members of the human innate immune system are different leukocytes such as monocytes, eosinophils, neutrophils, basophils, dendritic cells, and natural killer (NK) cells. Other non-cellular members of the innate immune system are the complement system and a large number of secreted cytokines as inflammatory response to any given trigger. Thus the innate immune system forms a complex and effective protective shield against infections but also malignant transformation, and cancer, respectively. Interestingly, several natural compounds like resveratrol strongly influence the immune response and e.g. modulate the activity of NK cells. Therefore, the modulation of the innate immune system by nutrition-derived compounds has an important and valuable impact on health. Due to the strong link between nutrition and cancer the field of nutritional immunology intensively investigates immune-modulating substances that are present or even enriched in foods for cancer prevention and treatment.

### NK cells and immune response

**Fig. 1:**
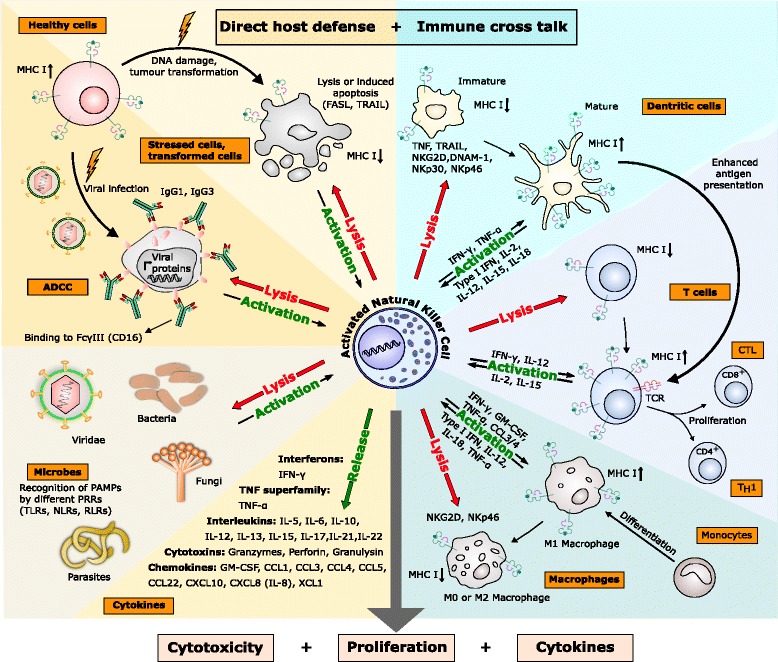
NK cells execute multiple tasks in innate immunity, they are i) responsible for direct defence of the host organism by checking for and eliminating stressed or transformed autologous cells with low MHC I levels, ii) execute ADCC in case of virus infected cells by binding IgG to FcγRIII (CD16) receptor, or iii) eliminate microbes by recognition of conserved structures with different PRRs. On the other side NK cells influence maturation and activation of other immune cells and e.g. can kill immature DCs or M0 and M2 macrophages and thus selectively let activated APCs present antigens to T cells in a controlled manner. Activated T cells can also be killed by NK cell-mediated lysis. NK cells therefore directly manipulate the adaptive immune response by influencing antigen presentation and quantity of other immune cells. Immune cross talk often implies bidirectional activation, which leads, like activating signalling in direct host defence, to enhanced proliferation, cytokine production, and cytotoxicity by increased expression of granzymes, perforin, and granulysin. Numerous cytokines can so be released by NK cells, primarily IFN-γ, TNF-α, and GM-CSF, but also many ILs and various inflammatory chemokines, which attract and traffic e.g. T cells, DCs, monocytes, eosinophils, basophils, or neutrophils. ADCC, antibody-dependent cell-mediated cytotoxicity; CCL, C-C motif ligand; CTL, cytotoxic T lymphocyte; CXCL, C-X-C motif ligand; DNAM-1, DNAX accessory molecule-1; FASL, fragment apoptosis stimulating ligand; GM-CSF, granulocyte macrophage colony-stimulating factor; IFN, interferon; IL, interleukin; MHC I, major histocompatibility complex I; MIP1, macrophage inflammatory protein 1; NK cells, natural killer cells; NKG2D, natural-killer group 2, member D; NKp30/46, natural killer cell p30/46-related protein; NLR, nucleotide oligomerization domain (NOD)-like receptor; PAMP, pathogen-associated molecular pattern; PRR, pattern recognition receptor; RLR, RNA helicase retinoic inducible gene I (RIG-I)-like receptor; TCR, T cell receptor; TLR, Toll-like receptor; TNF, tumour necrosis factor; TRAIL, TNF-related apoptosis-inducing ligand; XCL, X-C motif ligand

NK cells were first identified in 1975 by their ability to lyse cancer cells in vitro without prior immune sensitization [1, 2] and comprise about 15 % of all circulating lymphocytes [3]. Their main importance lies in early host defence against both allogenic and autologous cells after virus infection [4], infection with bacteria or parasites, or against malignantly transformed cells [5]. NK cell development primarily occurs in the bone marrow (BM) environment: they are derived from hematopoietic stem cells subsequently differentiating into common lymphoid progenitors, which finally develop into NK/T progenitors, from which NK cells are derived throughout life [6–8]. Their lineage development is characterized by the sequential acquisition of surface receptors and effector functions [9]. In addition to BM and blood, NK cells are also found in peripheral tissues including liver, peritoneal cavity, and placenta [10]. Human NK cells are broadly defined as CD3^−^ CD56^+^ (CD3: T-cell co-receptor; CD56: neural cell adhesion molecule (NCAM)) lymphocytes and are further distinguished into CD56_bright_ (~10 % of human NK cells) and CD56_dim_ (~90 % of human NK cells) NK cells. CD56_dim_ NK cells express high levels of Fcγ receptor III (FcγRIII, CD16) mediating antibody-dependent cell-mediated cytotoxicity (ADCC), and CD56_bright_ NK cells show less or no CD16 expression [11, 12]. To defeat their targets NK cells are, after prior activation by cytokines, capable of extravasation and infiltration into affected tissues [13, 14]. Target cell killing is executed through different mechanisms (Fig. 1). First, NK cells form so-called immune synapses (dynamic interface formed between a NK cell and a target cell). Second, NK cells release cytoplasmic granules, organelles containing proteins like perforin (Prf1), the saposin-like family member granulysin, and serin-proteases called granzymes like granzyme B (GzmB) to cleave e.g. several pro-caspases, which then are able to trigger apoptosis in the target cell [15, 16]. Furthermore, the expression of members of the tumour necrosis factor (TNF)-family like FAS ligand (FASL), TNF, and TNF-related apoptosis inducing ligand (TRAIL) are able to induce tumour-cell apoptosis upon formation of immune synapses. TRAIL can bind to several death receptors (DR), two of which are agonistic (DR4 (TRAIL-R1) and DR5 (TRAIL-R2)) and induce apoptosis, and two of which are antagonistic (decoy receptor 1 (DcR1, TRAIL-R3) and DcR2 (TRAIL-R4)) and cannot induce apoptosis. Another possibility to take action against target cells is the secretion of a number of effector cytokines such as interferon-γ (IFN-γ), granulocyte-macrophage colony-stimulating factor (GM-CSF), and interleukin (IL) like IL-5, IL-10, or IL-13 after reaching distinct stages of NK-cell differentiation (Fig. 1). In addition, NK cells secrete a variety of chemokines including chemokine C-C motif ligand 2 (CCL2, monocyte-chemoattractant protein (MCP)-1), CCL3 (macrophage inflammatory protein (MIP)-1α), CCL4 (MIP-1β), CCL5 (regulated upon activation, normal T-cell expressed and secreted (RANTES)), chemokine X-C motif ligand 1 (XCL1, lymphotactin), and chemokine C-X-C motif ligand 8 (CXCL8, IL-8) to colocalize with other immune cells like dendritic cells in areas of inflammation (Fig. 1) [17]. With a wide range of pattern recognition receptors (PRRs), different types of immune cells can specifically identify conserved pathogen-associated molecular patterns (PAMPs), which are exclusively present on microbes such as viruses, bacteria, parasites, and fungi. Members of the main PRR families are transmembrane Toll-like receptors (TLRs), C-type lectin receptors (CLRs), cytoplasmic nucleotide oligomerization domain (NOD)-like receptors (NLRs), and RNA helicase retinoic acid inducible gene I (RIG-I)-like receptors (RLRs). Thus, an intracellular signalling can be activated that subsequently induces expression of genes involved in inflammatory and/or immune response to recruit e.g. phagocytic cells and effector molecules to the site of infection. NK cells express different PRRs like TLRs, NLRs, and RLRs. They directly respond to PAMPs in an appropriate environment in the presence of cytokines like IL-2, IL-12, IL-15, or IL-18. Thus, activated NK cells produce IFN-γ, GM-CSF, or TNF-α, or release cytotoxic granules directed toward a target cell. Whether a NK cell remains silent or executes its killing capacity on malignant cells depends on the dynamic balance of stimulation events of two main structural classes of NK cell surface receptors, the killer cell immunoglobulin-like receptors (KIRs) and receptors of the C-type lectin-like family, which inhibit and/or activate signalling cascades (Fig. 1). Some of the human activating receptors like different KIRs or natural cytotoxicity receptors (NCRs) such as NKp30, NKp44, NKp46, and NKp80 transmit the activation signal via protein tyrosine kinase-dependent pathways. Therefore, different transmembrane adaptor proteins comprise one to three cytoplasmic immunoreceptor tyrosine-based activation motifs (ITAMs) consisting of a consensus amino-acid sequence with tyrosines and leucines [18]. After phosphorylation the ITAMs serve as docking sites for other kinases to further pass the signalling. Additional activating signals can also be mediated through receptors, which are noncovalently associated with other adaptor proteins, which contain no ITAM [19]. To antagonize NK cell activation, inhibitory surface receptors like different KIRs in humans are present, which act through protein tyrosine phosphatase-dependent pathways [20]. They harbour immunoreceptor tyrosine-based inhibitory motifs (ITIMs) in their cytoplasmic domains, which can recruit tyrosine phosphatases like Src-homology 2 domain (SH2)-containing SHP-1 or SHP-2. The equilibrium of the phosphorylation status of several signalling molecules which are targets for both members of the Syk-family of protein tyrosine kinases zeta-chain-associated protein kinase 70/SYC (ZAP70/SYC), SHP-1, SHP-2 protein phosphatases, and its shifting to the one side or the other is therefore crucial for NK cell behaviour. Ligands for the inhibitory receptors are polymorphic major histocompatibility complex (MHC) class I molecules. KIR receptors bind groups of HLA-A, HLA-B, and HLA-C alleles, while HLA-E is recognized by CD94-NKG2A.

### Cancer and NK cell activities

Some cancer cells lack or downregulate one or several MHC class I molecules and/or upregulate e.g. NKG2D ligands (NKG2DL) like the stress-inducible surface glycoproteins MHC class I-related chain A and B (MICA and MICB), and therefore provide no or not enough inhibitory stimulation [13, 21–23]. This so-called ‘missing-self’ recognition enables NK cells to detect and destroy transformed or allogenic cells while discriminating them from normal host cells (Fig. 1) [24]. Unfortunately, cancer patients frequently have functionally impaired NK cells and therefore a hindered antitumour immune response [25–27]. Therefore, the application of pharmacological compounds that enhance NK cell function and/or restore immune surveillance is part of current antitumour strategies and treatment regimes. Immunomodulatory drugs like thalidomide and lenalidomide augment cytotoxicity in multiple myeloma and increase the amount of peripheral blood NK cells [28, 29]. Chemotherapeutics like melphalan, etoposide, and doxorubicin, or the proteasome inhibitor bortezomib trigger the upregulation of activating ligands for the receptors NKG2D and DNAX accessory molecule-1 (DNAM-1) on multiple myeloma cells, thus sensitizing them to NK cell-mediated killing [30]. In line, bortezomib at low concentrations inhibits proliferation in hepatocellular carcinoma with simultaneously increased MICA/B expression [31]. Especially, a large number of cytokines such as IL-2, IL-10, IL-12, IL-15, IL-18, IL-21, IL-23, and type I interferons (IFN-α, IFN-β) are investigated for their modulation potential towards NK cell activity [32]. IL-2 and IL-15 are commonly needed to expand donor NK cells in vitro in adoptive transfer therapy to stimulate proliferation in the periphery [33]. This activation leads to high membrane-bound TRAIL expression when compared to unstimulated cells [34]. IL-2 is FDA approved for the treatment of metastatic renal cancer and advanced malignant melanoma [35]. Whereas long-term low-dose subcutaneous application of IL-2 seems to be associated with a tolerable side-effect profile, the systemic use of IL-2 in high doses can result in severe side effects like vascular leak syndrome (VLS) and other toxicities [36–38]. Of note, several natural compounds strongly influence the immune response and especially modulate the activity of NK cells while concurrently displaying favourable toxicity profiles. Resveratrol, daily administered to healthy volunteers in oral doses up to 5 g for a period of 29 days, was shown to be safe without serious adverse reactions, which was proved by clinical, biochemical, or hematologic analyses [39].

### Resveratrol a plant-based diet

**Fig. 2:**
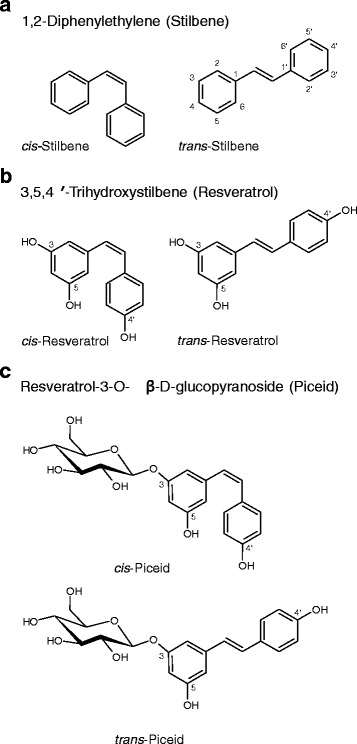
The parent compound of resveratrol is a trihydroxylated stilbene (**a**). Resveratrol exists in two isomeric forms, cis and trans (**b**). Its natural occurring glycosidic form is piceid (**c**) with a glucose molecule linked via a 3-O-β-D-glycosidic bond to cis- or trans-resveratrol

The naturally occurring lipophilic plant polyphenol resveratrol was first isolated in 1939 from the roots of the white hellebore *Veratrum grandiflorum O. Loes* by Takaoka [40]. Since then, resveratrol was extracted from over 100 different plants, some of which serve as common human dietary sources like grapes (wine, grape juice), peanuts, soy, hop, and berries like blueberries and cranberries. Resveratrol belongs to the polyhydroxystilbene subclass of plant polyphenols and exists as two isomers, cis-(Z) and trans-(E) (Fig. 2a and b). The styrene double-bond can undergo isomerization during UV irradiation from the trans- to the cis-form [41]. In the naturally occurring glycoside piceid a glucose moiety is linked to cis- or trans-resveratrol via a 3-O-β-D-glycosidic bond, so that also two piceid isomers exist (Fig. 2c). In plants resveratrol serves as a phytoalexin (plant antibiotic) produced in response to fungal infection, injury, or UV irradiation [42–45], especially in grapevines, pines, and legumes. Resveratrol gained public attention associated with the “French paradox”, a phrase describing the fact that the mortality rate from coronary heart disease (CHD) in France is lower than in the rest of Europe and the USA despite a diet traditionally rich in saturated fats and similar plasma cholesterol concentrations. Nevertheless, French mortality rates from CHD resemble more the ratios of Japan or China [46–48]. Corresponding data was acquired during the MONICA (Multinational MONItoring of trends and determinants in CArdiovascular disease) project organised by the World Health Organisation (WHO) in the 1980s to monitor cardiovascular diseases and to determine corresponding risk factors in 21 countries around the world. As possible explanation for this finding the consumption of red wine in France with its comparably high resveratrol content on a regular basis was suggested [49]. In fact, France had the highest per capita annual wine consumption worldwide during the period of data acquisition. Moreover, for resveratrol antioxidant [50, 51], anti-inflammatory [52], neuroprotective [53], antiproliferative [54, 55], and distinctive immunomodulatory properties were shown [56]. Further, multiple examples for antitumoural effects of resveratrol are described in literature and comprehensively summarized by Han and colleagues for different tumour types [57]. Recent publications describe e.g. a synergistic effect of resveratrol in combination with doxorubicin in vitro and in vivo in the treatment of different breast cancer cell lines (MCF-7 and MDA-MB-231) [58] or dose-dependent induction of apoptosis in colon cancer cell lines like SW620 and HepG2 cells [59, 60].

### Bioavailability, pharmacokinetics, and biological functions of resveratrol

Resveratrol is absorbed by intestinal trans-epithelial diffusion [61, 62]. In a clinical study by Walle et al. [63] at least 70 % of ^14^C - labelled resveratrol was taken up after oral administration. Further pharmacokinetic analyses revealed the highest resveratrol/metabolite levels 30 min after ingestion [64] with free resveratrol being present only to a small extent (1.7–1.9 %). Resveratrol-3-O-sulfate, resveratrol-4′-O-glucuronide, and resveratrol-3-O-glucuronide are the major plasma metabolites, accounting for 2.4-up to 13-fold greater C_max_ values in plasma than free resveratrol [65]. Almost 50 % of resveratrol and its metabolites are bound to plasma proteins like albumin and haemoglobin [66] as well as low density lipoproteins (LDL) [67, 68]. About 40–98 % of orally administered resveratrol is excreted into urine and faeces within 24 h [69]. Resveratrol first gained greater attention through its antioxidative activity against human LDL described in 1993 by Frankel et al. [51], thereby strengthening the “French paradox” hypothesis [46] via decreasing endothelial damage, which is pathophysiologically associated with cardiovascular disease. However, the antioxidant potential of resveratrol is less potent than that of quercetin or epicatechin, respectively flavonoids, which are more abundant in red wine than resveratrol [51]. Inhibition of platelet aggregation and eicosanoid synthesis by resveratrol due to decreased levels of thromboxane A2 (TxA2) via inhibition of cyclooxygenase-1 (COX1) was reported [70, 71]. This inhibiting property of resveratrol on cyclooxygenase activity plays a role in the production of pro-inflammatory molecules. In this context resveratrol acts as an anti-inflammatory molecule and was shown to reduce acute and chemically induced oedema [72, 73], lipopolysaccharide (LPS)-induced airway inflammation [74], and osteoarthritis [75]. Furthermore, resveratrol suppresses nuclear factor κ-light-chain-enhancer of activated B cells (NFκB)-activation [76–78], thus influencing gene transcription regulating immune and inflammatory responses [79]. Since 1997 it is known that resveratrol also bears an anticancer activity being active throughout the steps of tumour initiation, promotion, and progression in vitro as well as in vivo. Therefore, resveratrol was considered as a cancer chemopreventive agent [72]. Resveratrol also activates sirtuin 1 [80], which is responsible e.g. for the regulation of glucose and insulin production, fat metabolism and, notably, prolonged cell survival through negative regulation of the tumour suppressor p53 [81]. Moreover, resveratrol was also described to prevent dysregulation of gap junctional intercellular communication (GJIC) mediated by organic peroxids and environmental toxicants [82, 83]. For cancer but also other diseases alterations in the GJIC have been reported and seem to play a crucial role during malignant transformation and tumour promotion. Therefore, the protection of an impairment of the cellular GJIC adds another interesting aspect to the anticancer function of resveratrol [82, 83].

### Resveratrol and its interplay with NK cells

**Fig. 3:**
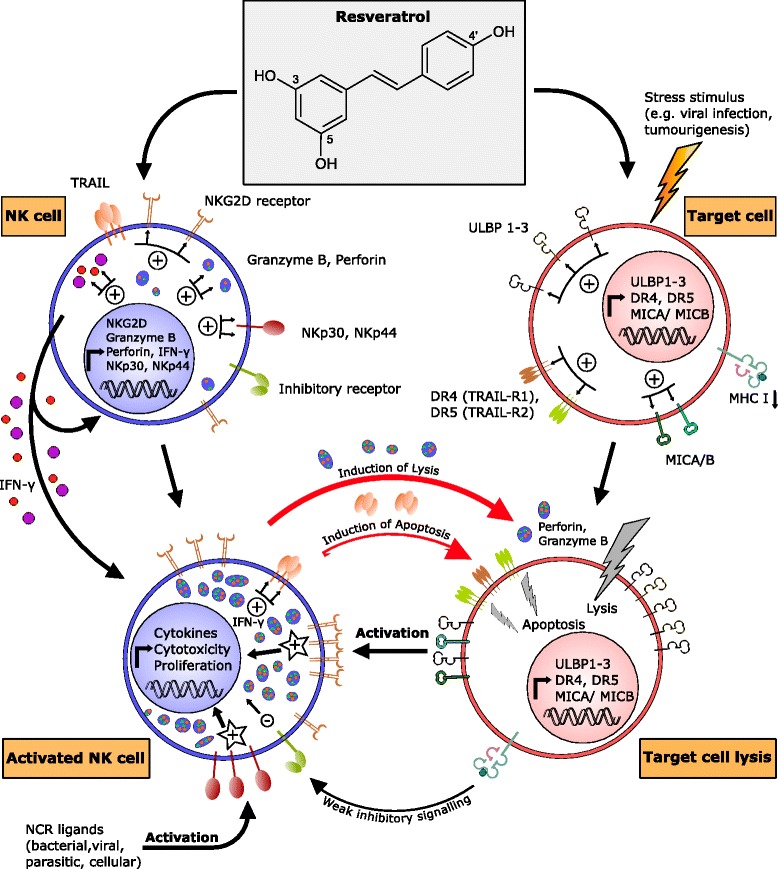
Resveratrol modulates the NKG2D receptor/NKG2D-L system by increasing expression in NK cells and in transformed target cells. Enhanced expression of NKG2D receptor and cytotoxins in NK cells together with upregulation of NKG2D ligands and DRs on target cell surface lead to enhanced killing efficacy. NK cells use two different mechanisms to kill the targets: i) by cytotoxic granule exocytosis ii) by induction of death receptor-mediated apoptosis. Increased IFN-γ production by resveratrol enhances TRAIL expression, which can facilitate apoptosis induction. Inhibitory signalling is often too weak to prevent NK cell killing due to downregulated expression of MHC I proteins in virus-infected or malignantly transformed cells. Further activating signals can provide NCR ligands of different origin. DR4/5, death receptor 4/5; MHC I, major histocompatibility complex I; MICA/B, MHC class I-related chain A/B; NK cells, natural killer cells; NKG2D, natural-killer group 2, member D; NKp30/44, natural killer cell p30/44-related protein; TRAIL, TNF-related apoptosis-inducing ligand; ULBP1-3, UL16 binding protein 1–3

Several studies demonstrated a direct influence of resveratrol on NK cells and their killing ability on different levels (Fig. 3). Resveratrol exerts simultaneous effects on NK cells and other immune cells like CD8^+^- and CD4^+^-T-cells [84]. Falchetti and colleagues exposed peripheral blood mononuclear cells (PBMCs) to different concentrations of resveratrol for a period of 18 h. After removing resveratrol, NK cell killing capacity of the PBMCs was tested against human immortalised myelogenous leukaemia K562 cells. The authors showed an increase of NK cell killing activity at low resveratrol concentrations ranging from 0.33 μM to 5.48 μM, with maximum activity at 1.31 μM. However, a dose-related inhibition of lytic activity was observed at high resveratrol concentrations of 21.92 μM and 87.68 μM. This finding was confirmed by Li and coworkers, who similarly demonstrated an inhibition of viability and increased apoptosis of NK cells upon incubation with high resveratrol concentrations (50 μM), whereas low concentrations from 1.56 μM to 3.13 μM resulted in upregulation of NKG2D and IFN-γ on mRNA as well as protein levels and an increased NK cell killing towards leukaemia K562 target cells (Fig. 3) [85]. These results suggest a concentration-dependent biphasic effect of resveratrol, which is explained by promoting cell apoptosis via caspase signalling pathway in high concentration ranges. This is supported by significantly reduced late apoptotic/necrotic cells after pretreatment with the caspase inhibitor z-VAD-FMK. The latter study further showed a higher cytotoxic susceptibility of human lymphoblastoid T cells (Jurkat cells) towards resveratrol when compared to NK cells. This was further corroborated by Lu and Chen, who reported a similar dose-dependent increase of cytotoxic NK cell killing activity also against tumour cell lines derived from solid tumours, e.g. HepG2 and A549 cells after pre-stimulation of immortalized NK cells (NK-92 cells) with resveratrol at low concentrations of 1.56, 6.25, and 12.5 μM. All effector to target ratios (1:1, 5:1, 10:1) showed similar effects with the highest enhancement of killing activity after pretreatment with 12.5 μM resveratrol for the 10:1 ratio [86]. The authors additionally demonstrated a dose-dependent upregulation of perforin expression and a dose-dependent phosphorylation of ERK-1/2 and JNK in resveratrol-stimulated NK-92 cells. ERK-1/2 and JNK have previously been shown to contribute to NKG2D-mediated cytotoxicity [87]. Using a murine acute pneumonia model to evaluate the anti-infectious properties of resveratrol, subsequently displayed an enhanced NK cell activity with an increased anticancer effect [88]. In the latter study resveratrol was intragastrically administered to rats for 3 days at 0.5 mg/kg body weight. The rats were subsequently intratracheally inoculated with *Serratia marcescens*, a common nosocomial pathogen, and monitored for 24 h. The resveratrol-treated group showed an increased alveolar macrophage (AM) infiltration, an elevated NK cell activity, and a decreased bacterial burden in the lungs of the infected animals, with a decreased mortality. Interestingly, isolated spleen NK cells of rats pretreated with resveratrol showed an enhanced killing efficacy against mouse ^51^Cr-labelled lymphoma YAC-1 target cells compared with spleen NK cells isolated from control rats treated with saline. In addition to the above-mentioned modes of action, resveratrol increases cell-surface expression of NKG2D ligands on human promyeloblastic leukaemia KG-1a cells, thus providing two complementary mechanisms to strengthen cytokine-induced killer cells (CIK, a mixed phenotype between T- and NK cells) killing properties directly and indirectly [89]. Stimulation of KG-1a cells with 25 μM resveratrol for 24 h rendered KG-1a cells susceptible to CIK-mediated cytolysis via an increase in cell-surface expression of NKG2D ligands and receptor DR4, coupled with a downregulation of cell-surface expression of DcR1 in KG-1a cells, and accompanied by activation of the TRAIL pathway [89]. Resveratrol is further capable of sensitizing cells of various cancer entities to TRAIL-induced apoptotic cell death such as neuroblastoma, medulloblastoma, glioblastoma, melanoma, T-cell leukaemia, pancreatic-, breast-, and colon cancer (Fig. 3) [90–92]. In this respect, resveratrol upregulates the agonistic receptors DR4 and DR5 in androgen-insensitive human prostate carcinoma cells PC-3 and DU-145 [93, 94], thus enhancing TRAIL sensitivity and possibly facilitating NK cell-mediated killing. Likewise, enhancement of DR4 and DR5 surface expression on TRAIL-resistant human prostate adenocarcinoma LNCaP cells with no difference for DcR1/2 after treatment with 10 μM resveratrol for 48 h was reported. Further, a dose-dependent activation of caspase-3 for resveratrol treatment alone, and caspase-8 activation for combined treatment with resveratrol and TRAIL was shown. For PC-3 prostate cancer cells similar results were obtained concerning increase of receptor expression of DR4 and DR5 for resveratrol treatment with 10 μM and 20 μM for 48 h, and caspase 3/8 activation for treatment with resveratrol (0-30 μM) and in combination with TRAIL (25 nM). Human 1205 LU metastatic melanoma cells show a resveratrol-dependent enhanced sensitivity to TRAIL through downregulation of the antiapoptotic proteins cFLIP and Bcl-xL [95]. Resveratrol also significantly enhances CD95L expression on HL60 human leukaemia cells and on T47D breast carcinoma cells after 24 h of treatment [96], which in addition facilitates NK cells to trigger signalling-dependent apoptosis. Nieswandt et al. showed a connection of platelet aggregation and the susceptibility of cancer cells to NK cell-mediated lysis [97]. In this respect, mouse and human cancer cells can activate platelets and their aggregation, which correlates with their metastatic potential [98, 99]. Due to tumour cell-platelet aggregation, circulating tumour cells (CTCs) can be coated by aggregated platelets and thus escape the immune response, which further facilitates metastasis. Interestingly, resveratrol mediates a dose-dependent inhibition of platelet aggregation via reduction of integrin gpIIb/IIIa on the platelet membrane, which acts as fibrinogen receptor involved in clot formation through the formation of bridges between platelets, and by reducing the production of TxA2, which activates further platelets and thus increases aggregation, through inhibition of COX1-dependent pathways [71]. In the field of NK cells resveratrol could further possess therapeutic potential in defeating aggressive NK cell leukaemias and lymphomas by inhibiting constitutively active signal transducers and activators of transcription 3 (STAT3) signalling, which was demonstrated in the work of Quoc Trung and colleagues in 2013 [100].

## Conclusion

### Modulation of NK cell activity by nutrition-derived compounds and the role of resveratrol

Historically, the impact of nutrition on immune function first came to light in 1810 when thymic atrophy in malnourished patients was described by J.F. Menkel [101]. Especially in the last decade, the field of nutritional immunology or immunonutrition has constantly been growing and established many molecular connections between dietary compounds and their influence on immune cells. Some of the different NK cell modulating compounds can occur in numerous plants with often very variable content like quercetin [102]. Since up to 70 % of the body’s immunocytes and over 90 % of all Ig-producing cells are located in the gut, which is therefore considered as the largest immune organ, the influence of nutrient compounds on human immunity by making first contact with the receptors of the immune cells in this area, is obvious [103, 104]. Moreover, the gut-associated lymphoid tissue (GALT), consisting of isolated and aggregated lymphoid follicles, is exposed to myriads of different food antigens and multiple microorganisms ingested with the daily diet. It is therefore predestinated for immunomodulation by nutrients e.g. through targeting PAMP receptors of innate immune cells. Immune-modulating molecules can be endogenous or exogenous, and share the ability to enhance or suppress immune responses. Many plant-derived secondary metabolites like flavonoids exhibit stimulating properties on NK cell activity. The flavonol quercetin enhances NK cell killing activity towards YAC-1 target cells [105]. Another study described an increased susceptibility of leukaemia K562, and gastric cancer SNU1 and SNU-C4 cells towards NK cell-mediated lysis through induced expression of different NKG2D ligands [106]. Myricetin from red wine and other foods showed similar stimulating effects towards K562 cells [107]. In the latter study, however, quercetin did not alter NK cell killing activity. Moreover, amentoflavone treated mice showed enhanced NK cell activity and ADCC. Furthermore, IL-2 and IFN-γ production was increased [108]. There are also examples of natural compounds, which are non-supportive for the immune system e.g. tangeritin, a flavone found in tangerine and other citrus peels, significantly reducing the number of lymphocytes in mice treated with 100 μM tangeritin in the drinking water for 4 weeks [109]. In another study, inhibitory effects of tangeritin on NK cell proliferation and differentiation with an ED_50_ (i.e., dose that is effective in half the cultures) between 1 μM and 10 μM, were observed [110]. In line with the immune-modulating properties of nutrition-derived compounds resveratrol has been described to affect NK cell function directly and indirectly on different levels. Resveratrol seems to enhance immune reactions e.g. via alteration of the expression of activating cell surface receptors like NKG2D on NK cells or via stimulation of the expression of their corresponding ligands on malignant cells as summarized in Fig. 3. This immune modulation is very interesting in the context of the further molecular properties of this natural compound. For resveratrol, a histone deacetylase (HDAC) inhibitory function in different human hepatoblastoma cells towards the classical HDACs (class I, II and IV) was detected. It was postulated, that HDAC inhibition mediated a dose-dependent reduction of cancer cell proliferation [111]. Interestingly, one preclinical approach for the activation of the NK cell population and a subsequent increased cancer cell killing is upregulation of the expression of the excitatory NKG2D ligands by HDAC inhibitors [23, 112]. In detail, Armeanu and colleagues demonstrated an enhancement of cell-surface expression of MICA/B in HepG2 and Hep3B hepatoma cells with no alterations of ULBP1-3 levels upon stimulation with the HDAC inhibitor valproic acid (VPA), which led to an enhanced NK cell-mediated killing [23]. Contrariwise, pretreatment of NK cells alone with classical HDAC inhibitors like VPA or suberoylanilide hydroxamic acid (SAHA) at therapeutic concentrations suppresses the cytolytic activity of NK cells [113]. Resveratrol is probably one of the most famous natural food ingredients due to its suspected ability to lower the incidence of coronary heart disease in people consuming wine with a high resveratrol content on a regular basis like in France, hence the name “French paradox”. Nonetheless, despite the beneficial properties, including antitumour activities of resveratrol, an important aspect remains to be discussed. Most studies and experiments concerning the immune-stimulatory activity of resveratrol were performed in vitro and ex vivo, respectively with supraphysiological concentrations. The content of resveratrol in different fruits, vegetables, and especially in processed foods as well as the bioavailability has to be taken into account while evaluating the biological activity of resveratrol. A study about the absorption of wine-related polyphenols including resveratrol in different matrices like wine, grape juice, and vegetable juice revealed maximum serum peak concentrations around 30 min after consumption with maximal serum concentrations of only 10–40 nM after consumption of 25 mg trans-resveratrol per 75 kg body weight (dissolved in 100 ml beverage, resulting in a concentration of 1.095 mM), which is considerably lower than the concentrations tested in vitro showing beneficial effects [64]. Accordingly, therapeutically required concentrations are not achievable without further dietary supplementation, which argues against an exclusive role of resveratrol in providing health-promoting properties by regular wine consumption alone. Resveratrol content of different French wines ranges from 0.6-6.8 mg/l (2.63-29.79 μM) [114], which is between 416- and 37-fold less than in the above mentioned absorption study. Taken together, resveratrol is a promising natural compound able to stimulate immune responses including an increase of the NK cell-mediated killing of (virus-) infected and malignantly transformed cells. Problems arise, however, in reaching pharmacologically effective systemic plasma concentrations, because of rapid phase II-metabolism and renal elimination. Nonetheless, suitable dietary intake of resveratrol could provide beneficial health effects.

## Abbreviations

ADCC: antibody-dependent cell-mediated cytotoxicity
APC: antigen presenting cell
BM: bone marrow
CCL: C-C motif ligand
CDS: cytosolic DNA sensor
CHD: coronary heart disease
CIK: cytokine-induced killer cell
CLR: C-type lectin receptor
COX1: cyclooxygenase-1
CTC: circulating tumour cell
CTL: cytotoxic T lymphocyte
CXCL: C-X-C motif ligand
DNAM-1: DNAX accessory molecule-1
DR4/5: death receptor 4/5
FASL: fragment apoptosis stimulating ligand
GALT: gut-associated lymphoid tissue
GJIC: gap junctional intercellular communication
GM-CSF: granulocyte macrophage colony-stimulating factor
GzmB: granzyme B
HDAC: histone deacetylase
IFN: interferon
IL: interleukin
ITAM: immunoreceptor tyrosine-based activation motif
ITIM: immunoreceptor tyrosine-based inhibitory motif
KIR: killer cell immunoglobulin-like receptor
LDL: low density lipoprotein
MHC I: major histocompatibility complex I
MICA/B: MHC class I-related chain A/B
MIP1: macrophage inflammatory protein 1
NCAM: neural cell adhesion molecule
NCR: natural cytotoxicity receptor
NK cell: natural killer cell
NKG2D: natural-killer group 2, member D
NKp30/44: natural killer cell p30/44-related protein
NKp30/46: natural killer cell p30/46-related protein
NLR: nucleotide oligomerization domain (NOD)-like receptor
PAMP: pathogen-associated molecular pattern
PBMC: peripheral blood mononuclear cell
Prfl: perforin
PRR: pattern recognition receptor
RANTES: regulated upon activation, normal T-cell expressed and secreted
RLR: RNA helicase retinoic inducible gene I (RIG-I)-like receptor
SAHA: suberoylanilide hydroxamic acid
STAT3: transducer and activator of transcription 3
TCR: T cell receptor
TLR: Toll-like receptor
TNF: tumour necrosis factor
TRAIL: TNF-related apoptosis-inducing ligand
TxA2: thromboxane A2
ULBP1-3: UL16 binding protein 1-3
VLS: vascular leak syndrome
VPA: valproic acid
WHO: World Health Organisation
XCL: X-C motif ligand
